# Department of Surgery

**Published:** 1899-01

**Authors:** L. A. Merillat, W. E. A. Wyman

**Affiliations:** Secretary of M’killip Veterinary College, Chicago; Milwaukee, Wis.


### DEPARTMENT OF SURGERY.

By L. A. Merillat, V.S.,
SECRETARY OF M'KILLIP VETERINARY COLLEGE, CHICAGO.

WITH THE COLLABORATION OF

W. E. A. Wyman, M.D.V., V.S.,
MILWAUKEE, WIS.,

AND OTHERS.

### On the Importance of Clinical Diagnosis.

The success of the veterinary surgeon depends on his knowledge of clinical diagnosis, provided he is otherwise equal to the task. The inferior diagnostician only succeeds so long as his competitors are his equals, otherwise he is or becomes a remarkable failure.

The clinical diagnosis of surgical diseases is difficult, although less so than of internal diseases. The innumerable and actually inexcusable mistakes, often the ruin of a young man and at times the beginning of the downfall of the old practitioner, are daily and conclusive proof. We simply refer to lameness. It is a standard fact that he who diagnoses lameness correctly invariably is or becomes a leading surgeon, provided he meets the demands of the public in other directions.

Certain operations are often condemned—operations of absolute value, such as low plantar neurectomy, cunean tenectomy, peroneal tenotomy, even the actual cautery—simply because the operator's diagnosis is open to decided criticism. Yet low plantar neurectomy in navicular disease is pronounced useless, cunean tenectomy spoken of as of little value in spavin, and peroneal tenotomy of no earthly use in stringhalt, the latter simply because with some surgeons any jerky motion of the leg passes as stringhalt. Therefore, these so-called failures depend primarily on a faulty diagnosis.

On the whole, it may be safely stated that the majority of practitioners studied clinical diagnosis at the expense of their customers. How many of those practising to-day, including the graduate of but three or four years' standing, enjoyed exhaustive, conscientious instructions in the clinical diagnosis of lameness? It is no exaggeration that able men of to-day never were given an opportunity to hear a lecture or see clinical demonstrations of even the most common surgical ailments. It is a feeling of relief to be able to state that this absolute farce so prevalent in the past and necessarily suicidal to a place of learning (?) is more or less being rapidly corrected in our veterinary colleges.

As previously stated, the veterinarian's foundation to success is laid in the college clinic. Here he is shown the intimacy existing between theory and practice; here theory, under proper guidance, finds or should find its practical application.

The clinical diagnosis of surgical diseases requires a thorough, and thus methodical, examination. The name of a disease is of secondary importance, but its cause or causes, their relationship to treatment, and finally the treatment, are the points to be ascertained. The eye, ear, and hands must be educated, not only to see, hear, and feel, but to execute their duties from the standpoint of the expert—that is, they must recognize the existing state and its relationship to the disease under observation; merely attending lectures and demonstrations will never complete the education of the various senses taxed in clinical diagnosis. Persistent, well-applied and properly directed efforts of a practical nature are essential.

The English-speaking student, unless a linguist, has no literature on the clinical diagnosis of any surgical diseases except, perhaps, the writer's own feeble effort to supply such a want on the clinical diagnosis of lameness. We earnestly hope that a work on clinical diagnosis is forthcoming, since it is nothing short of a necessity. It is regrettable that those most gifted and best equipped by reason of their superior knowledge on this subject are either too busy or too modest to create such a work.

Surgery not only being a science, but also an art, the intimacy between senses and hands is striking. After a certain state is recognized, correctly interpreted, the hands execute what ingenuity and experience dictate.

Energy, decided powers of observation, either congenital or acquired, are fundamental. Those talented find comparatively few obstacles, while the less fortunate ones need not despair, as continuous and well-applied efforts in the clinic will permit them to become able diagnosticians and neat operators.

W. E. A. Wyman, M.D.V., V.S.,
Milwaukee, Wis.

---

*To Fasten Labels on Tin Boxes.* Labels may be made to adhere firmly to tin ointment-boxes by first wiping the surface of the boxes with a weak solution of hydrochloric acid.

